# Epidemiological evidences on overdiagnosis of prostate and kidney cancers in Korean

**DOI:** 10.4178/epih/e2015015

**Published:** 2015-03-07

**Authors:** Jong-Myon Bae

**Affiliations:** Department of Preventive Medicine, Jeju National University School of Medicine, Jeju, Korea

**Keywords:** Prostatic neoplasms, Kidney neoplasms, Cancer screening, Incidence, Disease progression

## Abstract

**OBJECTIVES:**

The prostate specific antigen test is widely used as the main method of screening prostate cancer in Korea. Additionally, the use of ultrasound sonography may lead to overdiagnosis of kidney cancer as well as thyroid cancer. This study aimed to highlight epidemiological evidences regarding overdiagnosis of prostate and kidney cancers in Korean.

**METHODS:**

The annual trends of national incidence and mortality of prostate and kidney cancers provided by the Korean Statistical Information Service were evaluated.

**RESULTS:**

The rate of increase in the incidence of prostate and kidney cancer was 6 and 5 times higher than that of mortality between 2000 and 2011, respectively. Additionally, the age group showing the highest incidence in prostate cancer shifted from 85 years and older to 75-79 years.

**CONCLUSIONS:**

This evidence suggests that prostate and kidney cancers are overdiagnosed in Korea. Further research in this area, using national cancer registry databases, should be encouraged to prevent overdiagnosis.

## INTRODUCTION

Cancer screening, which reduces cancer mortality through early detection, inevitably leads to the problem of overdiagnosis [1]. Overdiagnosis is defined as ‘detection of cancers that would never have been found were it not for the screening test’ [2], and is regarded as a harmful aspect of screening testing because it results in unnecessary overtreatment [3]. In South Korea (hereafter Korea), concerns have recently been raised regarding the overdiagnosis of thyroid [4] and breast cancers [5].

A debate on overdiagnosis occurs when a cancer screening project is introduced, or new screening tests for early detection of cancer are developed [2]. In the West, there have been claims of overdiagnosis relating to thyroid, breast, prostate, and kidney cancers [1]; however, in Korea, where testing for prostate specific antigen (PSA) levels in blood is universal, an investigation of overdiagnosis of prostate cancer would provide important evidence for cancer management policies.

In addition, thyroid cancer shows the highest incidence among cancers in the Korean population [6], leading to claims of overdiagnosis due to expansion and supply of ultrasound testing devices [4]. However, ultrasound devices can increase the likelihood of discovering incidental cancer in the neck and in the abdominal organs [7]. Thus, one might suspect that kidney cancer is also currently being overdiagnosed. With respect to these questions, author aimed to investigate the epidemiological evidences on overdiagnosis of prostate and kidney cancers in Koreans.

## MATERIALS AND METHODS

The judgement criterion for the presence of overdiagnosis is that mortality does not change substantially compared to an in crease in incidence [2]. The annual trends of national incidence and mortality of prostate and kidney cancers provided by the Korean Statistical Information Service (KOSIS) [8] were evaluated.

## RESULTS

### Prostate cancer

**Figure 1.**
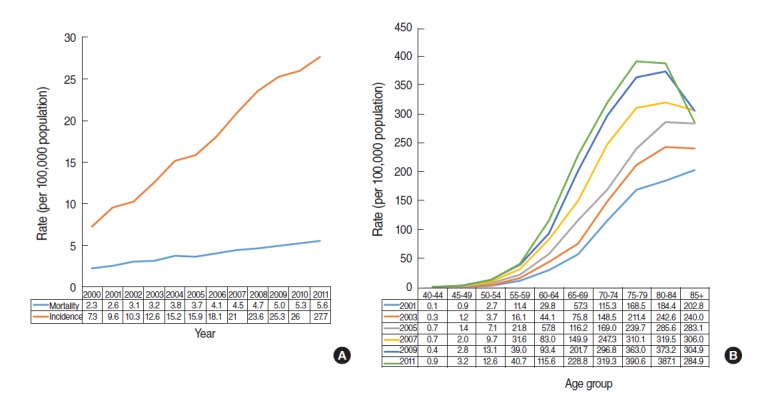
Prostate cancer in Korean men. (A) Age-adjusted incidence and mortality between 2000 and 2011. (B) Age-adjusted incidence in 2001, 2003, 2005, 2007, 2009, and 2011 by age group.

Welch and Black [1] presented evidence for prostate cancer overdiagnosis in that the incidence increased considerably immediately after an introduction of PSA screening; however, there was little change in mortality. There is similar evidence in relation to the overdiagnosis of thyroid cancer in Korea [7]. Figure 1(A) shows the annual incidence and mortality of prostate cancer provided by the KOSIS [8]. Compared with 2000, mortality in 2011 had increased by 3.3 (=5.6-2.3) deaths per 100,000 people, while prevalence increased by 20.4 (=27.7-7.3) cases per 100,000 people—a 6-fold increase (=20.4/ 3.3). This is the first piece of epidemiological evidences on the overdiagnosis of prostate cancer.

At the same time, Hilton et al. [9] reported that the age of diagnosis had decreased since the expansion of PSA testing. Accordingly, Figure 1(B) describes the incidence for different age groups of Korean males over the last 10 years. The age group with the highest incidence shows a shifting trend, from the over-85 group in 2001, to the 80 to 84 years group from 2003 to 2009, and the 75 to 79 years group in 2011. This is the second piece of epidemiological evidences on overdiagnosis. However, for the evidence to be more convincing, it is necessary to determine whether there has been a change in the distribution of disease stage at diagnosis [10].

### Kidney cancer

Approximately 10% of kidney cancers were incidental cancers in the early 1970s; however, with the development of various imaging technologies such as computer tomography and magnetic resonance imaging, this increased to approximately 60% in the 1990s [7]. Accordingly, the majority of kidney cancers are discovered incidentally while performing radiological tests for other purposes [11]. Compared with symptomatic tumors, incidental tumors are smaller, have favorable prognostic scores, and are less likely to metastasize [12]. As more incidental cancers are diagnosed, the incidence of kidney cancer will increase. Meanwhile, mortality is not expected to change, and even if it increases, the magnitude of change is not expected to be as large as that for incidence. This line of reasoning can explain the phenomenon in developed countries with a high economic level whereby the incidence of kidney cancer is high but mortality is low [13].

**Figure 2.**
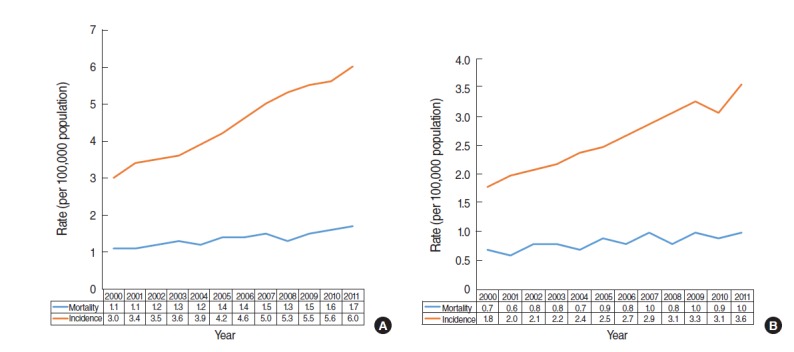
Age-adjusted incidence and mortality of kidney cancer between 2000 and 2011 in (A) all Koreans and (B) in Korean women.

With the rate of detection of incidental cancer increasing following the proliferation of radiodiagnostic machines, any large discrepancy between an increase in incidence and increase in mortality provides epidemiological evidence of overdiagnosis [1]. While mortality in kidney cancer doubled in the US between 1971 and 2010, incidence increased 5-fold [14]. In particular, a report showing a significant change in incidence supports a conclusion of overdiagnosis due to changes in the healthcare environment rather than in relation to age group or birth cohort over time [15]. Figure 2A shows the incidence and mortality trends of kidney cancer from 2000 to 2011 provided by the KOSIS [8]. Compared with 2000, mortality in 2011 had increased by 1.5-fold (=1.7/1.1), while incidence had doubled (=6.0/3.0); thus, there appears to be little difference. However, as mortality increased by 0.6 (=1.7-1.1) deaths per 100,000 people and incidence increased by 3.0 (=6.0-3.0) cases per 100,000 people, the rate of increase was 5 times (=3.0/0.6) higher for incidence compared with mortality. In other words, based on the difference in the slope of the curve in Figure 2, the gap between incidence and mortality is increasing with time to date.

Meanwhile, one of epidemiological characteristics of kidney cancer is that it is twice as common in males compared with females [16]. Figure 2B presents the pattern of increase for incidence and mortality in females. Over the same time-period, the rate of increase for incidence was 2.2 times (=2/0.9) higher than mortality for males, and 6.0 times (=1.8/0.3) higher for females. Given that thyroid cancer, as detected by neck ultrasound, is far higher in females [6], future research will be necessary to investigate whether the larger discrepancy shown for females with kidney cancer is related to the diffusion of ultrasound.

### Esophageal cancer

As mentioned above, if overdiagnosis of prostate cancer and kidney cancer results from expanded testing, other cancers that are the target of screening might also be overdiagnosed. In terms of national cancer screening, upper endoscopy for gastric cancer and mammography for breast cancer, at 70.9% each, are the most common clinical examinations [17]. With regards to upper endoscopy, the most commonly used method in screening, is it possible that esophageal cancer could be overdiagnosed given that the process involves transit through the esophagus to the stomach?

**Figure 3.**
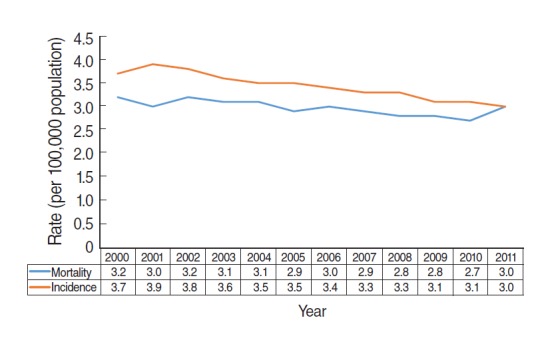
Age-adjusted incidence and mortality of esophageal cancer between 2000 and 2011 in the Korean population.

Figure 3 shows the annual incidence and mortality for esophageal cancer provided by the KOSIS [8]. Between 2000 and 2011, both incidence and mortality decreased or were unchanged. These data demonstrate that there was no overdiagnosis of esophageal cancer, despite the performance of upper endoscopies. Accordingly, overdiagnosis occurs when a ‘length time bias’ is introduced due to the expansion of screening programmes for indolent cancers [2,3].

## DISCUSSION

There is a need for caution when interpreting the phenomenon of incidence-mortality discrepancy given as epidemiological evidences of overdiagnosis in Figure 1(A) and Figure 2(A). The reason, in terms of prostate cancer, can be explained by increased screening provision, improved diagnostic sensitivity, and changes in risk factors [19]. The discrepancy for kidney cancer can also be explained by increased testing, development of new screening methods such as genetic testing, changes in terms of risk factors such as quitting smoking, development of effective treatments, and medical care provision systems [16,18].

Given the volume of epidemiological evidence relating to overdiagnosis, natural history studies of the pertinent cancers are required to determine its extent [2,16]. In other words, there is a need to calculate the scale of overdiagnosis in order to evaluate the risk-benefit payoff of screening [2]. Also, evidences relating to the phenomenon of the smaller size and lower stage of cancer at diagnosis in cases of overdiagnosis should be provided in the national cancer registration data managed by the Korea Central Cancer Registry (KCCR) [2,9]. With research limitations due to the reinforcement of personal information protection [20], author recommends the KCCR to take the lead in using the cancer registration data, constructed with the purpose of calculating national cancer statistics, to produce useful data for the development of cancer management policies. In addition, multifaceted research activities by healthcare workers to minimize overdiagnosis are also necessary [2].
