# Supplementary material for: Epidemiological evidences on overdiagnosis of prostate and kidney cancers in Korean
Source: Epidemiol Health. 2015 Mar 7;37:e2015015. doi: 10.4178/epih/e2015015 (PMC4398977; doi:10.4178/epih/e2015015)
Supplement: Supplementary file 1 [file epih-37-e2015015-supplementary.pdf]

<Brief Communication>

한국인 전립선 및 신장암의 과진단에 대한 역학적 근거

Epidemiological Evidences about Overdiagnosis of Prostate and Kidney Cancer in Korean

Abstract

Purpose: Prostate specific antigen test is widely diffused as the main method of screening prostate cancer in Korea. And diffusion of ultrasound sonography may cause overdiagnosis of kidney cancer as well as thyroid cancer. The aims were to suggest epidemiological evidences about overdiagnosis of prostate and kidney cancer in Korean.

Methods: The annual trends of national incidences and mortalities of prostate and kidney cancers offered by Statistics Korea were evaluated.

Results: The increasing velocities of incidences in prostate and kidney cancers were 6 and 5 times higher than of mortality between 2000 and 2011, respectively. And the age group showing the highest incidence in prostate cancer was shifted from 85 years and older into 75-79 years.

Conclusions: These facts suspected the overdignosis of prostate and kidney cancers in Korean. The academic activities for creating the related evidences using nationwide cancer registry database should be encouraged to control of overdiagnosis.

Key words: Prostate neoplasms, Kidney neoplasms, Cancer screening, Incidence, Disease progression

1. 서론

암의 조기발견을 통한 암사망을 줄이려는 암 검진 (cancer screening)은 필연적으로 과진단 (overdiagnosis)의 문제를 야기시킨다[2]. 과진단이란 '평생동안 몰랐을 암을 검진으로 알아낸 경우'로 정의하고 있는 바[2], 불필요한 과치료 (overtreatment)를 유발한다는 점에서 검진의 위해 (harm)로 보고 있다[3]. 우리나라에서 최근 갑상선[4]과 유방[5]에 있어 과진단의 우려가 제기되었다.

과진단은 암검진사업이 도입되거나, 새로운 암검진용 검사가 개발될 때 논란이 생긴다[2]. 서구에서는 갑상선암, 유방암, 전립선암, 신장암 등에서 과진단이 있다는 주장이 있어 왔는데[1], 혈중 전립선특이항원 (Prostate Specific Antigen, 이하 PSA)검사가 보편화된 국내 상황에서 전립선암에 대한 과진단의 여부를 확인하는 것은 암관리정책상 중요한 근거가 된다.

또한 전국민 암발생통계에 있어 갑상선암의 발생률이 1위인 것[6]은 초음파 검사기기의 보급 및 확산에 따른 과진단의 결과라는 주장이 제기되었다[4]. 그런데, 초음파 기기는 목뿐만 아니라, 복부의 장기들에 대하여도 우연 암 (incidental cancer)을 발견할 기회를 높일 수 있다[7]. 그렇다면 신장암 (Kidney cancer)에 대하여도 진료현장에서 과진단이 일어나고 있는 것은 아닐까라는 의문도 생기게 된다. 이상의 의문제기에 따라, 한국인에 있어 전립선암과 신장암에 대한 과진단의 역학적 근거들을 알아보고자 한다.

2. 본론

가. 전립선암

Welch & Black [1]이 전립선암의 과진단을 주장한 근거로 제시한 것은 검진이 확산된 직후부터 발생률이 크게 증가하지만, 사망률 변동은 미약하다는 것이다. 이는 국내 갑상선암의 과진단에 대한 주장근거로도 활용되었다[7]. Fig. 1 (A) 는 통계청 포탈[8]에서 제공하는 전립선암의 발생률과 사망률을 연도별로 나타낸 것이다. 2000년도에 비하여 2011년의 사망률은 십만명당 3.3명(=5.6-2.3) 증가한 반면, 발생률은 십만명당 20.4명(=27.7-7.3)으로 6배 (=20.4/3.3) 더 증가한 것은 전립선암 과진단의 첫 번째 역학적 근거가 된다.

한편, Hilton et al. [9]는 PSA 검사가 확산된 이후 진단시 연령이 낮아짐을 보고하였다. 이에 Fig. 1(B) 는 지난 10년간 한국 남성에 있어 연령대별 발생률을 살펴본 것이다. 발생률이 최고가 되는 연령군이 2001년에는 85세 이상군, 2003-2009년에는 80-84세 군, 2011년도는 75-79세 군으로 이동하는 양상을 보이고 있다. 이것이 과진단의 두 번째 역학적 근거인 것이다. 그러나 진단시 병기 (stage)의 분포에서 변화가 있는 가를 같이 보아야만 더 설득력 있는 근거가 될 것이다[10].

나. 신장암

전체 신장암 중 우연 암이 차지하는 정도가 1970년 초에 약 10% 정도였지만, 그 이후 CT, MRI 등의 다양한 영상기기기 개발되면서 1990년대에서는 약 60% 로 증가하였다[7]. 따라서 대부분의 신장암은 다른 목적으로 영상의학 검사를 하는 과정에서 우연하게 (incidentally) 발견되는 경우이다[11]. 임상증상에 따라 의심하여 알아낸 경우 (symptomatic

tumors)보다 우연 암은 크기가 더 작고, 예후 점수도 더 양호하며, 전이도 더 적게 하는 것으로 알려져 있다[12]. 따라서 우연 암을 많이 진단할수록 신장암의 발생률은 증가하며, 사망률은 변화가 없거나 증가해도 발생률처럼 큰 변동은 보이지 않을 것으로 예상할 수 있다. 이에 따르면 경제수준이 높은 선진국일수록 신장암 발생률이 높지만 사망률은 낮은 현상을 설명할 수 있다[13].

이처럼 영상진단 의료기기의 보급 이후 우연 암이 많아지면서 발생률과 사망률간의 증가 폭에서 큰 간격을 보인다면, 이 또한 과진단이 있다는 역학적 근거가 된다[1]. 미국의 경우 1971년을 기준으로 2010년도 사망률이 2배 증가한 반면, 발생률은 5배 증가하였다[14]. 특히 발생률 변동에 있어 연령이나 출생코호트보다는 시기 (period)별로 유의한 변화가 있었다는 보고[15]는 보건의료 환경변화에 따른 과진단 결과임을 지지하는 소견이다. 통계청 포탈[8]에서 제공하는 신장암의 2000-2011년도 발생률과 사망률 추이를 Fig. 2(A)로 나타내었다. 2000년도를 기준으로 2011년도 사망률이 1.5 배 (=1.7/1.1) 증가하고 발생률은 2배 (=6.0/3.0) 로 나와 별 차이가 없어 보인다. 그렇지만 사망률 증가 폭이 십만 명 당 0.6명 (=1.7-1.1), 발생률이 3.0명 (=6.0-3.0)으로써, 사망률에 비하여 발생률의 증가 속도가 5배 (=3.0/0.6) 더 높다. 다시 말해서 Fig. 2의 곡선 기울기가 달라서 최근으로 올수록 발생률-사망률 간격이 더 크게 벌어지고 있는 것이다.

한편 신장암은 여자보다 남자에서 2배 더 높다는 역학적 특성이 있어[16], 여성의 발생률-사망률 증가 양상을 살펴본 것이 Fig. 2(B)이다. 같은 기간 사망률에 비하여 발생률 증가 속도에 있어 남자는 2.2 배 (=2/0.9), 여자는 6.0배 (=1.8/0.3)로 나왔다. 목 초음파로 알게 되는 갑상선암은 여자가 월등히 높다는 점[6]에서 신장암 여자에서 큰 간격을 보이는 것이 목 초음파 시행과 연관된 결과인가에 대한 추후 연구가 필요하겠다.

다. 식도암

국가암검진에 있어 가장 수검율이 높은 검진법은 70.9%를 보인 위암 검진을 위한 상부내시경검사 (upper endoscopy)와 유방암 검진을 위한 유방촬영술이다[17]. 앞서 언급처럼 전립선암과 신장암의 과진단은 검사의 보급확대에 따른 것이라면, 암검진 대상이 되는 다른 암들도 과진단이 생기느냐의 의문이 생긴다. 가장 수검율이 높은 상부내시경검사는 식도를 거쳐 위로 진행하는 검사 과정을 고려할 때, 식도암에 대하여 과진단이 유발될 가능성은 없는가?

Fig 3.은 통계청 포탈[8]에서 제공하는 식도암의 발생률과 사망률을 연도별로 나타낸 것이다. 2000년도를 기준으로 2011년도까지 발생률과 사망률 모두 감소하거나 변동이 없다. 이 소견은 상부내시경검사를 시행함에도 불구하고 식도암에 있어 과진단이 없음을 알려준다. 따라서 진단을 받은 이후 진행속도가 지체 (indolent)되는 암이면서, 암검진사업이 확산되어 기간차이 바이어스 (length time bias)가 개입될 때에 과진단이 일어나는 것이다[2,3].

3. 결론

Fig. 1(A)과 Fig. 2(A)에서 과진단의 역학적 근거로 제시한 발생률-사망률 괴리 현상에 대하여는 해석상 주의를 요한다. 그 이유는 전립선암에 있어서 검진 보급률 증가, 검사법의 민감도 향상, 발생위험 요인의 변동 등으로도 설명할 수 있기 때문이다[19]. 신장암에 있어서도 관련 검사의 시행률, 유전자 검사 같은 새로운 검사법의 개발, 금연 등의 발생위험요인의 변동, 효과적인 치료법 개발, 의료서비스 전달체계 등으로도 설명할 수 있기 때문이다[16,18].

이렇듯 과진단을 의심하는 역학적 근거들이 있으면, 그 크기를 알아낼 수 있도록 해당 암의 자연사 (natural history) 연구가 필요하다[2,16]. 다시 말해서 검진의 이득에 비하여 위해가 얼마나 큰 가를 판단하기 위해서 과진단의 규모를 산출할 필요가 있는데, 이는 중앙암등록본부가 관리하는 암등록자료를 활용하면 가능하다[2]. 또한 과진단이 있을 경우 진단시 암의 크기가 줄어들고, 병기 (stage)가 낮아지는 현상[9]에 대하여도 중앙암등록본부가 관리하는 전국민 암등록자료에서 해당 근거들을 생성할 수 있도록 해야 할 것이다[2]. 따라서 개인정보보호 강화로 연구에 제한이 있는 상황에서[20], 중앙암등록본부는 국가암통계 산출 목적으로 구축하는 암등록자료를 활용하여 암관리정책에 필요한 유용한 근거들을 주도적으로 산출해 주기를 권고한다. 이외에도 과진단을 최소화하기 위한 보건의료인의 다각적인 연구활동이 요구된다 [2].

감사의 글

이 논문은 2015학년도 제주대학교 학술진흥연구비 지원사업에 의하여 연구되었음

References

1. Welch HG, Black WC. Overdiagnosis in cancer. J Natl Cancer Inst 2010;102:605-613.

2. Bae JM. Overdiagnosis: Epidemiologic concepts and estimation 2015;37:e2015004.

3. Ilic D, Neuberger MM, Djulbegovic M, Dahm P. Screening for prostate cancer. Cochrane Database Syst Rev 2013;1:CD004720.

4. Ahn HS, Kim HJ, Welch HG. Korea's thyroid-cancer "epidemic"--screening and overdiagnosis. N Engl J Med 2014;371:1765-1767.

5. Bae JM. On the benefits and harms of mammography for breast cancer screening in Korean women. Korean J Fam Pract 2014;1:1-6. (Korean)

6. Jung KW, Won YJ, Kong HJ, Oh CM, Lee DH, Lee JS. Cancer statistics in Korea: incidence, mortality, survival, and prevalence in 2011. Cancer Res Treat 2014;46:109-123.

7. Ng CS, Wood CG, Silverman PM, Tannir NM, Tamboli P, Sandler CM. Renal cell carcinoma: diagnosis, staging, and surveillance. Am J Roentgenol 2008;191:1220-1232.

8. Statistics Korea. Korean statistical information service. National cancer statistics [cited 2015 Jan 30]. Available from: http://kosis.kr/statisticsList/statisticsList_01List.jsp?vwcd=MT_ZTITLE&parentId=A#SubCont

9. Hilton WM, Padalecki SS, Ankerst DP, Leach RJ, Thompson IM. Temporal changes in the clinical approach to diagnosing prostate cancer. J Natl Cancer Inst Monogr 2012;2012:162-168.

10. Li J, German R, King J, Joseph D, Thompson T, Wu XC, et al. Recent trends in prostate cancer testing and incidence among men under age of 50. Cancer Epidemiol 2012;36:122-127.

11. Krajewski KM, Giardino AA, Zukotynski K, Van den Abbeele AD, Pedrosa I. Imaging in renal cell carcinoma. Hematol Oncol Clin North Am 2011;25:687-715.

12. Rabjerg M, Mikkelsen MN, Walter S, Marcussen N. Incidental renal neoplasms: is there a need for routine screening? A Danish single-center epidemiological study. APMIS 2014;122:708-714.

13. Znaor A, Lortet-Tieulent J, Laversanne M, Jemal A, Bray F. International Variations and Trends in Renal

Cell Carcinoma Incidence and Mortality. Eur Urol 2014. doi: 10.1016/j.eururo.2014.10.002.

14. Leveridge MJ, Bostrom PJ, Koulouris G, Finelli A, Lawrentschuk N. Imaging renal cell carcinoma with ultrasonography, CT and MRI. Nat Rev Urol 2010;7:311-325.

15. Tyson MD1, Humphreys MR, Parker AS, Thiel DD, Joseph RW, Andrews PE, et al. Age-period-cohort analysis of renal cell carcinoma in United States adults. Urology 2013;82:43-47.

16. Cairns P. Renal cell carcinoma. Cancer Biomark 2010;9:461-473.

17. Suh M, Choi KS, Lee YY, Jun JK. Trends in Cancer Screening Rates among Korean Men and Women: Results from the Korean National Cancer Screening Survey, 2004-2012. Cancer Res Treat 2013;45:86-94.

18. Etzioni R, Gulati R, Mallinger L, Mandelblatt J. Influence of study features and methods on overdiagnosis estimates in breast and prostate cancer screening. Ann Intern Med 2013;158:831-838.

19. Zhu Y, Wang HK, Qu YY, Ye DW. Prostate cancer in East Asia: evolving trend over the last decade. Asian J Androl 2015;17:48-57.

20. Bae JM. Global trends in the use of nationwide big data for solving healthcare problems. J Korean Med Assoc 2014;57:386-390. (Korean)
